# HLA-G DNA sequence variants and risk of perinatal HIV-1 transmission

**DOI:** 10.1186/1742-6405-3-28

**Published:** 2006-10-23

**Authors:** Felix O Aikhionbare, K Kumaresan, Falah Shamsa, Vincent C Bond

**Affiliations:** 1 Department of Medicine, Morehouse School of Medicine, Atlanta, GA 30310, USA; 2 Department of Community Health and Preventive Medicine, Morehouse School of Medicine, Atlanta, GA 30310, USA; 3 Department of Microbiology/Biochemistry/Immunology, Morehouse School of Medicine, Atlanta, GA 30310, USA

## Abstract

**Background:**

*HLA-G *gene is a non-classical MHC class 1 molecule that is highly expressed in the trophoblast at the maternal-fetal interface. In an attempt to elucidate possible immunological mechanisms facilitating protection of infants born to human immunodeficiency virus type (HIV-1) infected mothers, we have been studying genetic variations in the coding and untranslated regions of HLA-G antigen between HIV-1-infected mothers and their infected or uninfected infants. This study investigated whether HLA-G DNA sequence variants are associated with perinatal HIV-1 transmission.

**Results:**

Genomic DNA samples were obtained from a nested case-control study of 34 mother-child pairs co-enrolled in a cohort of the Perinatal AIDS Collaborative Transmission Study in New York. The samples were from two groups predominantly of African-American and Hispanic origin: In the first group, both mother and child were HIV-1-infected; in the second group, only the mother was infected while the child remained uninfected. Genotyping of *HLA-G *gene were performed on the extracted DNA from peripheral blood mononuclear cells using PCR based sequencing and restriction fragment-length polymorphism analyses.

Among the studied *HLA*-*G *exons, dissimilarities in HLA-G DNA sequence variants between the HIV-1 non-transmitting mother child pairs were mostly observed in exon 8-3'-untranslated region at nucleotide positions T3742A, C3743T, G3777C (*P *= 0.001). Non-transmitting HIV-1 mother child pairs exhibited dissimilarities at nucleotide position C3743T allele with decreased risk of perinatal HIV-1 transmission, compared with HIV-1 transmitting mother-child pairs carrying this allele (odds ratio 0.02 [95% confidence interval 0.00–0.15] *P *= 0.00001). In addition, heterozygous dissimilarities at nucleotide positions C634G and 714 insT/G in the 5'-upstream regulatory region were observed between the mother child pairs of the HIV-1-non-transmitting group while homozygous similarities of C634C, and either 714insG/G or mother-child pairs with similar 714insT/G were observed among the transmitting group in the same region.

**Conclusion:**

This study identified new variants in the *HLA-G *gene and provides further evidence that dissimilarities in the HLA-G DNA sequence variants could influence the transmission of HIV-1 from infected mothers to their infants.

## Background

Mother-to-child HIV transmission (MTCT) can occur during pregnancy, labor and postnatally through breastfeeding [1,2]. In developed countries, MTCT has decreased to approximately 1 or 2% after implementation of universal prenatal HIV counseling and testing, antiretroviral prophylaxis, elective cesarean delivery and avoidance of breastfeeding [3]. Antiretroviral therapy for infected pregnant women has significantly reduced the risk of MTCT in developing countries. However, factors primarily related to logistics, costs and access have limited the impact of these interventions in developing countries. Although it remains important to continue to pursue strategies to make effective antiretroviral interventions available in developing countries, an efficacious vaccine would surely be highly valuable for reduction of MTCT and certainly would be the best strategy for reducing infection spread worldwide among adults. The development of an effective vaccine would be greatly assisted by better understanding of the molecular genetic mechanisms of transmission, including for example, if the host gene immune responses involving the chemokines [4,5], and HLA polymorphisms are involved in host-HIV-1 interactions.

Human leukocyte antigen G (HLA-G), a non-classical class 1 MHC gene, is highly expressed in trophoblast at the maternal-fetal interface. The unique expression of this gene in the trophoblast at the maternal-fetal interface indicates a critical role for this locus in human pregnancy. Past studies [6-8] suggest that *HLA-G *gene protects the trophoblast from maternal T-cell and natural killer cell (NK) mediated lysis. However, association of this *HLA-G *gene with HIV-1 infection or transmission is yet to be fully determined. Several lines of evidence suggest that fetal or newborn alloimmune responses directed at maternal HIV-1-infected cells or at free virus bearing maternal MHC determinants may account for some children remaining uninfected [9-11]. A study by Arthur *et al*., [12] showed that class 1 MHC antigens were present on the envelope of HIV-1 and that the antisera to these proteins precipitated intact virions [7]. Chan *et al*., [13] observed that immunization with purified class 1 HLA molecules could protect macaques from challenge with cell-free virus expressing the class 1 *HLA *peptides [9]. During mother-to-child transmission of HIV-1, it is unclear whether free HIV-1 virions or HIV-1-infected cells of maternal origin or both are present in the maternal MHC antigens. It is possible that either fetal or newborn anti-MHC or alloreactive T cell responses could protect the infant against infection transmitted from the mother. However, such mechanisms would only be operative if there were some degree of HLA-G sequence variants between the mother and the child. The effects would depend on mechanisms and changes in the function that resulted from the HLA-G sequence variants. Moreover, it could reasonably be expected that any protection provided may correlate with the extent of HLA discordance between the mother and the child as previously reported [9-11]. This study was designed to examine variants in the *HLA-G *gene that may play a role in perinatal HIV-1 transmission.

## Methods

### Study participation

A nested case-control study designed with subjects and controls were selected from enrolled participants that were previously followed as a part of Perinatal AIDS Collaborative Transmission Study (PACTS), a multi-center cohort study of vertical HIV transmission, funded by Centers for Disease Control and Prevention (CDC), which enrolled 2,665 HIV-positive mother-infants between 1986 and 1998 (360 HIV-1-infected infants). In this study, only participants enrolled in New York City were part of the selection of 34 mother-child pairs. Generally, PACTS cohorts collected early neonatal PCR data (< 48–72 hours after birth) and classified the perinatally HIV-1-infected infants in the cohort as: 1) intrauterine infection (DNA PCR positive < 48 hours) and 2) presumed peripartum infection (intrapartum and/or very late interuterine). The study protocols have been approved by institutional review boards at the respective participating sites including the CDC. Written informed consent were obtained from mothers who participated in this study.

### Participation criteria

Blood samples were obtained from infants who were at least after 2 months of age. Children known to have been breast-fed or whose HIV-1 status could not be determined were excluded. Also, all blood samples were from mother-child pairs who were not treated with antiretroviral drugs. The majority of samples included in this study were African American (59%) and Hispanic (29%). The mean birthweight among the infant participants were 2715 grams in the non-transmitting mother-child pairs and 2607 grams (*p *= 0.3) in transmitting mother-child pairs and the mean maternal CD4^+ ^T-lymphocyte count were 579 copies/mL and 488 copies/mL (*p *= 0.2) in the non-transmitting and transmitting groups respectively. Preterm delivery of the infants (< 37 weeks) were 29% (4/14) among the HIV-1-non-transmitting mothers and 30% (6/20) (*p *= 0.8) among the HIV-1-transmitting mothers. The CD4^+^:CD8^+ ^ratio was 0.9 [(0.61), 21.4% (3/14)] and 0.59 [(0.36) 5% (1/20)] (*p *= 0.5) among the non-transmitting and transmitting mothers respectively. In addition, rupture of membranes during delivery was 50% (7/14) among the HIV-1-non-transmitting and 55% (11/20) (*p *= 0.9) among the HIV-1-transmitting mothers. Mothers diagnosed with AIDS before delivery were 21.4% (3/14) among the non-transmitting group and 25% (5/20) (*p *= 0.8) among transmitting group.

### Study protocol

Genomic DNA samples were isolated from the blood drawn within 48 hours to 18 months after birth from the participated infants for HIV-1 PCR-based tests. Children enrolled in this study were tested again at months 1, 2, 4, and 6 and at a month interval thereafter. The cohorts followed all infected children and examined them at regular intervals according to the standard protocols: children were considered infected if they were HIV-1-seropositive, confirmed by western blot at ≥18 months of age, had two or more positive PCR tests or viral cultures at any age, had an AIDS-defining illness, or if they died with HIV-related condition [14].

### Sample source and DNA extraction

Extracted DNA were obtained from blood samples of 34 mother-child pairs, which included 20 HIV-1-infected mother-child pairs, and 14 infected mother-uninfected child pairs. Briefly, blood samples were collected in vacutainer collection tubes containing EDTA anticoagulant. Plasma and peripheral blood mononuclear cell (PBMC) fractions were prepared by Ficoll-Hypaque centrifugation. Cells in the PBMC fraction were washed in phosphate buffered saline (PBS). The PBMCs were lysed and genomic DNA were purified using the QIAamp blood PCR Kit (Qiagen, Chatsworth, CA), and quantified by UV absorption spectrometry. DNA was stored at -20°C.

### Amplification of HLA-G exons, intron, 5'URR and 8-3'UTR

Isolated genomic DNA samples from HIV-1-infected mother/child pairs were amplified using *HLA-G *allele-specific primers as previously described [7,15] for exons 3, and 4. Extensive care was taken to design primers for 5'untranslated regulatory region (5'URR) and primers spanning exons 6, 7 and 8-3' untranslated regions (8-3'UTR) using previously described nucleotide sequence of HLA-6.0 by Geraghty *et al*. [16]. Primers for 5'URR, (*5hlag1F*: *5'*-*GGGTTTCTC CCTGGTTTCTC-3' *(forward) and *3hlagex1R 5-CGAGGAGGGGTTGAGACC *-3' (reverse)), generated a 550-bp fragment as a part of 5'URR that spanned exon 1. Primers for 8-3'UTR, (*5hlaf6/7*: *5'-TTCCTCTAGGACCTCATGGCC-3' *(forward) and *3hlagex8 5'-AGGAAAGGTG ATTGGGGAAG-3' *(reverse)), generated a 590-bp fragment that spanned exons 6, 7, intron 7, exon 8 and a part of the 3'-UTR. The conditions for the amplification reactions for exons 3, 4 were as previously described [15] and the conditions for the amplification reactions for 5'URR and 8-3'UTR fragments, were as follows: 100 ng of DNA, 1 μM of each primer: 200 μM of each dNTP (Promega, Madison, WI) and 0.5 U *Taq *polymerase in 10× buffer (Fisher Scientific, Suwanee, GA). The 5'URR and 8-3'UTR PCR procedure was as follows: an initial denaturation step at 95°C for 5 min, and amplification for 35 cycles at 94°C for 1 min, 60°C for 1 min, 72°C for 1.30 min, followed by a final extension step at 72°C for 10 min, except, for 5'URR, the annealing was 64°C for 45 sec. PCR products were electrophoresed on 1% agarose gel.

### Sequencing and statistical analysis

All PCR products were purified using Qiagen gel extraction Kits (Chatsworth, CA). Sequencing was performed in both directions by Big Dye Terminator v.3 (Applied Biosystems) on an ABI 3100 Automated sequencer (Applied Biosystems), using PCR primers for all studied exons and internal sequencing primers specifically for 5'URR (*5HLAGPRO *5'*-GGCTCTCAGGG TCTCAGGCCCCAC*-3'(forward) and *HLAGEX1REV 5'-GGCCGTTTCCCTCCTGAC-3' *(reverse)). Sequencing results were analyzed using nucleotide-nucleotide BLAST searches. Also, sequences were aligned with previously published nucleotide sequences of Geraghty *et al*.,[16]. Restriction analysis was used to validate all sequence variants including the amplified PCR products spanning exons 6, 7 and 8 including intron 7 and part of 3-UTR that was digested with *Pst *I enzyme according to the manufacture's instructions (Promega). The digested products were subjected to 2% agarose gel electrophoresis and detected by staining with ethidium bromide. To assess differences between proportions in each of the HLA-G variant within each studied exon among HIV-1 non-transmitting and transmitting mother-child pairs, Fisher' exact test was applied with corresponding two-sided *p*-values, odds ratio (OR) and 95% confidence intervals (CIs). To assess overall difference between the exons, Pearson Chi-square test was used with respect to frequencies of the HLA-G DNA sequence similarities and dissimilarities between HIV-1 non-transmitting and transmitting mother-child pairs within and among exons and results were declared significant at an α = 0.05 using the SAS release 9.1.

### Definitions of similarity/dissimilarity HLA-G DNA sequence variant

Mother-child pairs were considered similar in HLA-G DNA sequence variant if both the mother and child were homozygous for that allele, heterozygous for the same variant within each of the studied exons, with reference to previously published HLA-G DNA sequence by Geraghty *et al*.,[16]. Mother-child pairs who shared only one variant that is heterozygous, or pairs in which the child was homozygous for that allele while the mother was not, was considered as dissimilarity of the HLA-G DNA sequence variants. Furthermore, mother-child pairs was defined as a HLA-G DNA sequence variant dissimilar if no shared HLA-G DNA sequence variants were identified between the mother and the child within the studied exons of the *HLA-G *gene.

## Results and discussion

**Figure 1:**
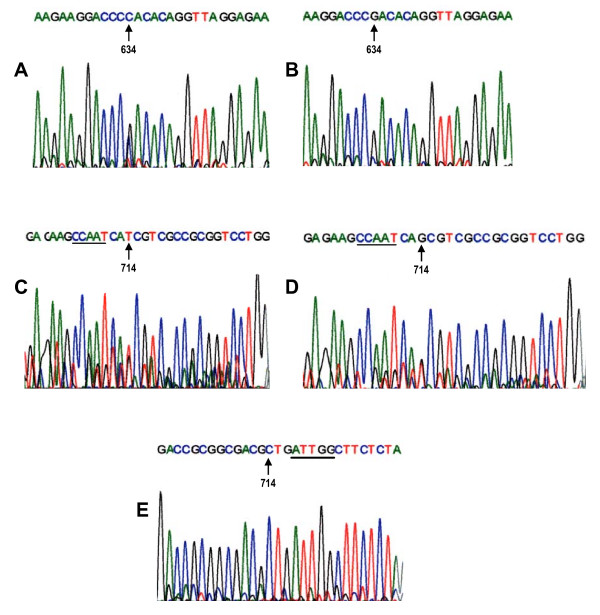
Sequence electropherograms obtained from HLA-G DNA 5' untranslated regulatory region showing polymorphisms at nucleotide positions 634 and 714. **A-B**: Reverse strand sequences and arrows indicating a nucleotide change of C→G observed between mother-child pairs among the HIV-1 non-transmitting group. **C-D: **Forward strand sequences showing insertions of either T or G. **E**: Reverse strand sequence and underlined is the *CCAAT box*. Interestingly, dissimilarities of the 714insT/G were observed between the mother child pairs of the HIV-1-non-transmitting group while, either 714insG/G or mother-child pairs with similar nucleotide changes were observed among the transmitting group.

**Figure 2:**
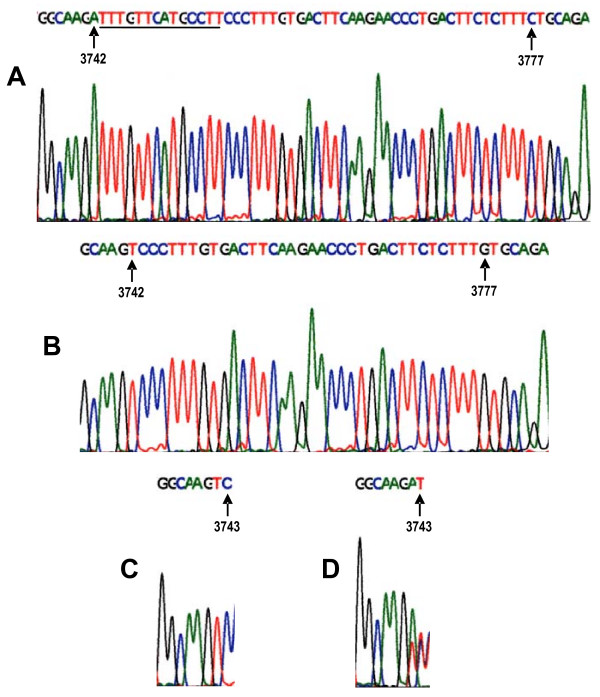
Sequence electropherograms obtained from 8-3' untranslated region showing polymorphisms at nucleotide positions 3742, 3743 and 3777. **A-B: **Forward strand sequences and arrows indicating nucleotide changes of T→A and G→C. Underlined is the +/-14bp. **C-D: **Sequences showing a nucleotide change of C→T indicated by arrows. Notably, C3743T sequence variant was observed at a high dissimilarity between mother-child pairs among HIV-1 non-transmitting groups.

Thirty-four samples of mother-child pairs (68 samples) were selected as described above, which included 14 pairs of HIV-1-infected mothers and their uninfected children and 20 pairs of mothers and children who were HIV-1-infected. Analysis of the HLA-G DNA sequences of exons 2, 3, 4, 6, 7, a part of 8-3'UTR and a part of 5'URR spanning exon 1 from these samples was performed. All sequences and positions were referenced with the wild-type HLA-G sequence of Geragthy *et al*., [16]. Twenty-one HLA-G sequence variants were observed as shown in Table 1. Seven out of 21 (33%) of the observed variants were previously described [7,8,14,18,19]. Fourteen out of 21 (67%) variants, (G634C, 714insT, C1486G, C1486A, G2575A, T2577A, C2614A, C2622A, 3401insC, G3579A, G3585T, C3619G, T3742A, and C3743T) (Table 1; Figures 1 and 2), were not previously reported.

**Table 1:** Variations in HLA-G DNA sequence observed among HIV-1-transmitting and non-transmitting mother-child pairs.

**Exons**	**Codons**	**Nucleotide positions **	Nucleotide substitutions*	**Amino acid changes **
5'URR	---	634 C/G	C→G	None
5'URR	---	714 ins T,G	insT,G	None
2 α-1-domain	31	1074 A/T	A→T	Thr-to-Ser
2 α-1-domain	57	1154 G/A	G→A	Pro-to-Pro
3 α-2-domain	93	1486 C/G	C→G	His-to-Asp
3 α-2-domain	93	1486 C/A	C→A	His-to-Asp
3 α-2-domain	93	1488 C/T	C→T	None (His)
3 α-2-domain	107	1528 G/A	G→A	Gly-to-Arg
3 α-2-domain	110	1537 C/A	C→A	Leu-to-Ile
3 α-2-domain	130	1597 del C	del C	Leu→frameshift
4 α-3-domain	256	2575 G/A	G→A	Arg-to-Lys
4 α-3-domain	257	2577 T/A	T→A	Tyr-to-Asn
4 α-3-domain	269	2614 C/A	C→A	Pro-to-His
4 α-3-domain	272	2622 C/A	C→A	Leu-to-Met
Intron 6		3401 ins C	ins C	None
Intron 7		3579 G/A	G→A	None
		3585 G/T	G→T	None
		3619 C/G	C→G	None
8 (3'UTR)		3742 T/A	T→A	instability of mRNA
8 (3'UTR)		3743 C/T	Del 14 bp	instability of mRNA
8 (3'UTR)		3777 G/C	G→C	instability of mRNA

*Polymorphisms are indicated with reference to the wild-type HLA 6.0 sequence previously published by Geraghty (1987).

A heterozygous dissimilarity at nucleotide position C634G was observed between the pairs of mothers and children among the HIV-1-non-transmitting group, while homozygous similarity at nucleotide position C634C was observed among transmitting mother-child pairs in the 5'URR of the *HLA-G *gene (Figure 1A and 1B). Furthermore, a dissimilar insertion of either G in mother to T in child or T in mother to G in child at nucleotide position 714 was found among the HIV-1 non-transmitting mother-child pairs, while mother-child pairs with similar 714insT/G or G/G were observed among the transmitting group (Figure 1C,D and 1E).

**Figure 3:**
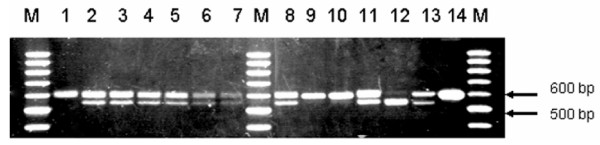
Representatives of digested PCR products of exons 6/7 and exon 8-3'UT region of HLA-G DNA obtained from blood samples, using restriction enzyme *Pst I*. M; 100-bp DNA marker, Lane 1; uncut PCR product, lane 2–7 three HIV-1-transmitting mother-child pairs (lane 2 and 3 mother-child pair, lane 4 and 5 mother-child pair; 6 and 7 mother-child pair); lane 8–13; three non transmitting mother-child pairs (lane 8 and 9 mother-child pair, lane 10 and 11 mother-child pair; 12 and 13 mother-child pair), lane 14; uncut PCR product.

In addition, analyses of DNA variants in exon regions 2, 3, 4, 6, 7 and 8-3'UTR of the *HLA-G *gene were performed to elucidate the association of the polymorphisms with HIV-1 transmission and results are shown in Table 1. There were no statistically significant associations of HLA-G gene variants in exons 3, 4, 6 and 7 with HIV-1-perinatal transmission among our studied samples (Table 2). In contrast, there was a statistically significant association of dissimilarity of the HLA-G DNA sequence variants among our studied exon 8-3'UTR samples (Table 2; Figure 2A,B,C and 2D) at nucleotide positions T3742A, C3743T, G3777C (*P *= 0.001) with decreased risk of perinatal HIV transmission. Dissimilarities in the HLA-G exon 8-3'UTR at nucleotide position C3743T was highly frequent among the non-transmitting mother-child pairs, 85.7% (12/14-were dissimilar) than the transmitting mother-child pairs 14.3% (2/14-were dissimilar) with decreased risk of perinatal HIV-1 transmission (OR 0.02 [95% CI, 0.00–0.15] *P *= 0.00001). Validation of the amplified PCR products spanning HLA-G exons 6, 7, and 8-3'UTR by RFLP, using *Pst *I restriction enzyme resulted in 85.7% (12/14) of the mother-child pairs showing dissimilarities of banding profiles among the HIV-1-non-transmitting group and 90% (18/20) similarity of banding profiles among the HIV-transmitting mother-child pairs (Figure 3).

**Table 2:** HLA-G DNA sequence variant dissimilarities/similarities between Mother-child pairs and risk of perinatal HIV-1 transmission

HLA-G Mutation Position		N = 20 HIV-1 Transmitting group	N = 14 HIV-1-non-Transmitting group	*P-Value *(2-sided)	*OR (95%CI)*
**Exon-2**					
Codon 31	Dissimilar	2 (10.0%)*	2 (14.3%)*	0.987	0.67 [0.08–5.39]
	Similar	18 (90.0%)	12 (85.7%)		
Codon 57	Dissimilar	8 (40.0%)	13 (92.9%)	0.003	0.05 [0.00–0.47]^9^
	Similar	12 (60.0%)	1 (7.1%)		
**Exon-3**					
Codon 93	Dissimilar	4 (20.0%)	6 (42.9%)	0.290	0.33 [0.07–1.53]
	Similar	16 (80.0%)	8 (57.1%)		
Codon 107	Dissimilar	1 (5.0%)	0 (0.0%)	0.806	2.23 [0.09–58.81]
	Similar	19 (95.0%)	14(100.0%)		
Codon 110	Dissimilar	5 (25.0%)	3 (21.4%)	0.866	1.22 [0.24–6.23]
	Similar	15 (75.0%)	11 (78.6%)		
Codon 130	Dissimilar	1 (5.0%)	0 (0.0%)	0.806	2.23 [0.09–58.81]
	Similar	19 (95.0%)	14 (100.0%)		
**Exon-4**					
Codon 256	Dissimilar	10 (50.0%)	2 (14.3%)	0.060	6.0 [1.06–34.00]
	Similar	10 (50.0%)	12 (85.7%)		
Codon 257	Dissimilar	10 (50.0%)	3 (21.4%)	0.150	3.67 [0.78–17.25]
	Similar	10 (50.0%)	11 (78.6%)		
Codon 269	Dissimilar	5 (25.0%)	0 (0.0%)	0.174	10.3 [0.52–203.07]
	Similar	15 (75.0%)	14 100.0%)		
Codon 272	Dissimilar	0 (0.0%)	2 (14.3%)	0.359	0.12 [0.01–2.75]
	Similar	20 (100.0%)	12 (85.7%)		
**Intron 6**					
3401	Dissimilar	0 (0.0%)	0 (0.0%)	undefined	undefined
	Similar	20 (100.0%)	14 (100.0%)		
**Intron 7**					
3579	Dissimilar	2(90.0%)	5 (35.7%)	0.090	0.2 [0.03–1.24]
	Similar	18 10.0%)	9 (64.3%)		
3585	Dissimilar	9 (45.0%)	6 (42.9%)	0.820	1.09 [0.28–4.32]
	Similar	11 (55.0%)	8 (57.1%)		
3619	Dissimilar	11 (55.0%)	6 (42.9%)	0.727	1.63 [0.41–6.46]
	Similar	9 (45.0%)	8 (57.1%)		
**Exon 8-3UTR**					
3742	Dissimilar	4 (20.0%)	10 (71.4%)	0.004	0.1 [0.02–0.49]
	Similar	16 (80.0%)	4 (28.6%)		
3743	Dissimilar	2 (10.0%)	12 (85.7%)	0.00001	0.02 [0.00–0.15]
	Similar	18 (90.0%)	2 (14.3%)		
3777	Dissimilar	5 (25.0%)	9 (64.3%)	0.03	0.19 [0.04–0.82]
	Similar	15 (75.0%)	5 (35.7%)		

*Percentage of similarity and dissimilarity; N, number of pairs in the given group;

Data from 34 mother-child pairs with HLA-G DNA sequence variants as compared to the previously published wild-type HLA 6.0 sequence by Geraghty (1987). Mother-child pairs matching with the same HLAG DNA sequence variants at the above HLA-G sequence positions are considered similar; Mother with a mismatched HLAG DNA sequence variants at one or more of these positions with the child is considered dissimilar.

This study suggests the association of the *HLA-G *sequence variants with increased or decreased risk of perinatal HIV-1 transmission. All mutations observed in this study are present in the HLA-G mRNA isoforms and these mRNA isoforms are abundant transcripts in the placental tissue [7]. Previously, we have reported the association of a conservative mutation in HLA-G exon 2 (which encodes for the *alpha-1*-domain), (OR, 0.5 [95% CI, 0.00–0.47] *p *= 0.003) codon 57 with increased risk of perinatal transmission of HIV-1 [9]. Although, the mutation in exon 2 region was considered as a silent mutation, a study by Duan *et al*., [17] has demonstrated that a silent mutation effects human dopamine receptor D2 stability and synthesis of the receptor. In contrast, a study by Matte *et al*., [18] that investigated *HLA-G *gene concordance/discordance in African population of MTCT found no association of the mutation in exon 2 region at codon 57 with perinatal HIV-1 transmission. The striking difference in the results may be explained by the population structure used in their study (90% of the subjects in their study were of Shona ethnicity, which may represent a homogenous ancestral group or considered an isolated population). In general, homogeneous populations have relatively low frequencies of genetic variability than the heterogeneous populations and certain mutations may not play a role in disease protection in isolated populations compared to mixed populations. Furthermore, the apparent discrepancies between Matte *et al*., [18] study and our previous results could be further attributed to the definition of concordance and discordance. Nevertheless, this study data suggests that *HLA-G *gene mutations in the 5'URR alone or in combination with exon 8-3'UTR among HIV infected mother/uninfected child pairs that exhibited mismatching in the HLA-G DNA sequence may be associated with decrease in the risk of mother-child transmission of HIV-1. Mutations in *HLA-G *gene, particularly in 3' UTR region, may enhance the reduction of the expression of *HLA-G *gene and hence play a role in the *in*-*utero*-HIV infectivity. A study has demonstrated that 14-bp deletion-insertion polymorphisms in the 3' UT region of the *HLA-G *gene influences HLA-G mRNA stability [19]. Other studies have shown that down regulation of the surface expression of *HLA-G *gene by viral immunoevasion of the placental MHC class I molecules like herpes simplex and cytomegalovirus results in viral replication and infection [20,21].

Surprisingly, we did not observe any previously described polymorphisms by Ober [22] and Matte [23], except one polymorphism reported by Hviid [8] at 714insG in the portion of the studied sequence of the untranslated regulatory and full length of exon 1 regions of the *HLA-G *gene. Nevertheless, 98–99% of the same regions in our study matched with the previously published sequence of that region by Geraghty *et al*., [16]. We observed six and four nucleotide sequence variations within exon 3 and exon 4 regions respectively. This is a striking difference between this study and previously reported 31 variations in exon 3 by van der Ven *et al*., [15,24]. However, the number of variations in exon 3 region was similar to those reports by Ishitani *et al*., [25] and Matte *et al*., [23]. Of note is that all the observed nucleotide changes in exon 4 region resulted in amino acid changes and three of the six observed nucleotide changes in exon 3 region resulted in amino acid change as well. No nucleotide variation was observed in exons 6 and 7 regions. An insertion of C at position 3401 in intron 6 region and three nucleotide changes, G3579A, G3585T, and C3619G, in intron 7 were observed in our samples. In exon 8-3' UTR, we observed four nucleotide changes including the 14-bp insertion/deletion. To our knowledge, these nucleotide changes in introns 6, 7 and the two nucleotide changes at 3742 and 3743 positions of 8-3'UTR (Tables 1, 2) have not been previously reported. The polymorphisms at nucleotide positions 3742 and 3743 that were observed in some sequences of our studied samples with the 14-bp deletions, may be considered as a precursor to the occurrence of the 14-bp deletion in the *HLA-G *gene and could possibly influence mRNA stability.

Interestingly, the dissimilarity of the HLA-G sequence variants at nucleotide position C634G and 714insT/G observed between the mother and child of HIV-1-non-transmitting group may be in linkage disequilibrium with another variant that confers low risk of HIV perinatal transmission. Although we do not have functional data to correlate this assumption, these polymorphisms identified in 5' URR of *HLA-G *gene in this study, may play an important role in the pre-transcription due to the location of these variants, which are up and downstream of the CCAAT box respectively. In addition, the 714insT or G is closely flanked by an interferon response factor-1 binding motif at the upstream regulatory element of *HLA-G *gene. Taken together, the data from this study fits the "Trojan exosome hypothesis" to some degree, which predicts relatively efficient retroviral transmission between individuals who are histocompatible and relatively inefficient retroviral transmission between individuals who are histoincompatible [26]. Given that *HLA-G *gen*e *is primarily expressed during fetal life, it could be envisaged that during gestation, a HIV-1-infected mother could transmit the virus to her fetus. This intrauterine exposure to HIV-1 virus may be a powerful selective force that could result in a polymorphism advantage at this HLA-G locus of fetuses, which can be presented with a wider variety of foreign peptides to the T-cell receptors. As a result, fetuses with a high similarity of HLA-G sequence variants with their mothers may be more susceptible to intrauterine HIV-1 infection compare to fetuses with HLA-G sequence variants that are relatively dissimilar but unlikely to be recognized as foreign by the maternal immune system. This deduced scenario is similar to the previously hypothesized scenario by van der Ven and Ober regarding HLA-G allogenic response [15].

## Conclusion

Based on these results, it is increasingly clear that HLA-G variants in exon 8-UTR, either alone or in combination with exon 2 codon 57 that was previously reported [9] and the 5' UTR could influence the risk of MTCT of HIV-1 infection. In addition, it is tempting to speculate that these observed HLA-G sequence variants may play a role in the *in-utero *HIV-1 transmission, even in the presence of other recognized risk factors [27-30]. However, the limited sample size of this data set suggests that the results should be interpreted cautiously. Future studies are required in a larger population of samples to determine the full impact of these HLA-G variants in perinatal HIV-1 transmission.

## Declaration of competing interests

The author(s) declare that they have no competing interests.

## Authors' contributions

FOA conceived, designed the study and carried out the molecular genetic studies, participated in the sequence alignment and drafted the manuscript. KK participated in acquisition of data, sequence alignment, drafted and reviewed the manuscript. FS performed the statistical analysis. VB participated in the design and helped to review the manuscript. All authors read and approved the final manuscript.
